# Supplementary material for: Age‐specific and compartment‐dependent changes in mitochondrial homeostasis and cytoplasmic viscosity in mouse peripheral neurons
Source: Aging Cell. 2024 Jun 17;23(10):e14250. doi: 10.1111/acel.14250 (PMC11464114; doi:10.1111/acel.14250)
Supplement: Supplementary file 1 — Movies S1–S6. [file ACEL-23-e14250-s001.zip › Movie S1-S6.docx]

**Movie** **1**: Representative time-lapse movie of mitochondria in axons of DRG neurons obtained from young (P92) and old (P676) mice and stained with MitoTracker Deep Red. Width: 73 μm. Movie plays at 20 frames per seconds and represents 2 min of real-time. Scale bar: 5 μm.

**Movie** **2**: Representative time-lapse movie of mitochondria from young (P72) and old (P624) MitoMouse sciatic nerve axons. Width: 64 μm. Movie plays at 20 frames per seconds and represents 4:26 min of real-time. The ‘Smooth’ filter in Fiji/ImageJ was applied for presentation purposes. Scale bar: 5 μm.

**Movie** **3**: Representative time-lapse movie of GEM particles in the cell body of a cultured DRG neuron. Movie plays at 20 frames per seconds and represents 30 seconds of real-time.

**Movie** **4**: Representative time-lapse movie of GEM particles in the axon of a cultured DRG neuron. Width: 55 μm. Movie plays at 20 frames per seconds and represents 30 seconds of real-time. The ‘Smooth’ filter in Fiji/ImageJ was applied for presentation purposes.

**Movie** **5**: Representative time-lapse movie of mitochondrial transport in MFCs acquired from axons of neurons in which the somal compartment was either left untreated (top movie) or was treated with sorbitol (middle movie) or PEG (bottom movie). Width: 80 μm. Movie plays at 20 frames per seconds and represents 1 min of real-time. The ‘Smooth’ filter in Fiji/ImageJ was applied for presentation purposes.

**Movie** **6**: Representative time-lapse movie of mitochondrial dynamics in MFCs acquired from neuronal cell bodies. Top movie: untreated control; middle movie: cell body treated with sorbitol; bottom movie: cell body treated with PEG. Movie plays at 20 frames per seconds and represents 1 min of real-time. In the cell bodies treated with crowding agents, the mitochondria appear more clustered and less dynamic.
