# Supplementary material for: Age‐specific and compartment‐dependent changes in mitochondrial homeostasis and cytoplasmic viscosity in mouse peripheral neurons
Source: Aging Cell. 2024 Jun 17;23(10):e14250. doi: 10.1111/acel.14250 (PMC11464114; doi:10.1111/acel.14250)
Supplement: Supplementary file 2 — Appendix S1. [file ACEL-23-e14250-s002.docx]

**Supplementary Figure 1**


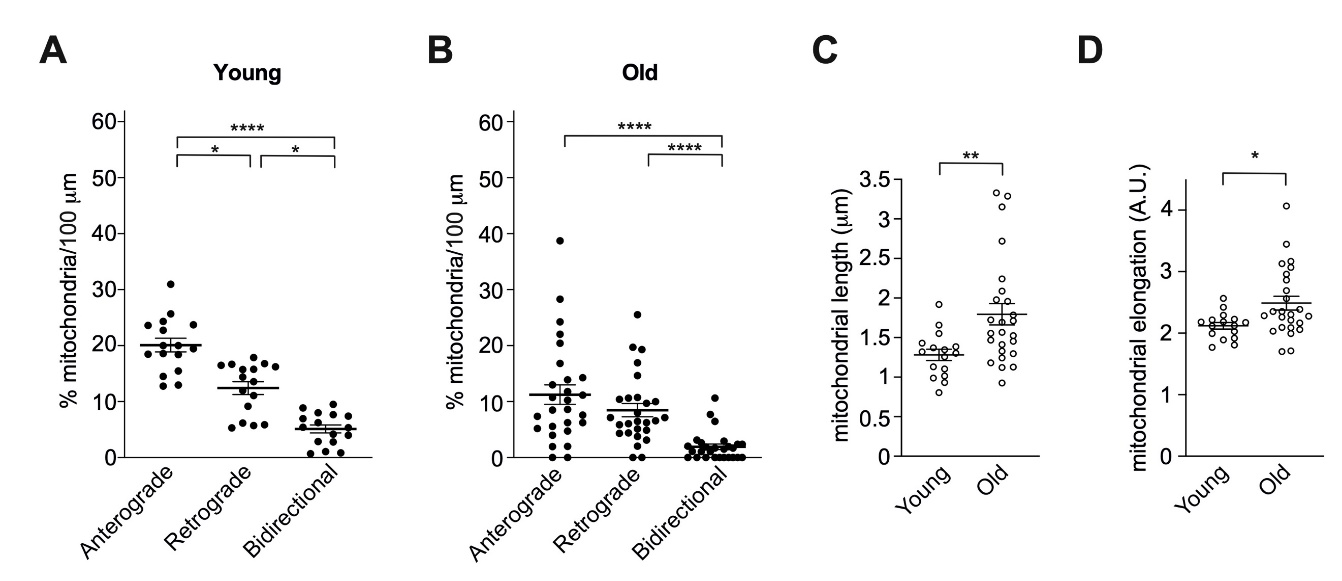


**Figure S1**

Proportion of motile mitochondria displaying anterograde, retrograde and bidirectional motility in young **(A)** and old **(B)** DRG neurons *in vitro* (relative to Figure 1C). A general decrease of motility is observed during age. **(C-D)** Quantification of mitochondrial length and elongation (see Methods) in young and old cells. Individual data points represent fields of view (FOV) imaged (1-3 axons/FOV). Data are shown as mean ± SEM. Kruskal-Wallis with Dunn’s multiple comparisons test (A-B) and Mann-Whitney test (C-D). * p < 0.05; ** p < 0.01; **** p < 0.0001.

**Supplementary Figure 2**

**
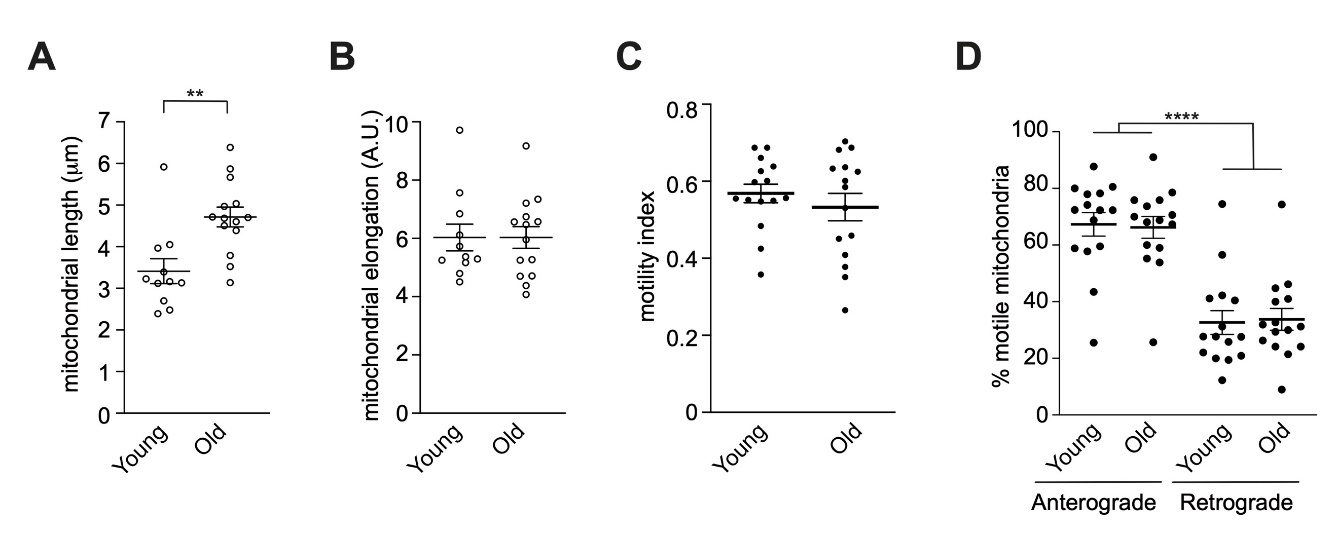
**

**Figure S2**

**(A-B)** Quantification of mitochondrial length and elongation (see Methods) in young and old sciatic nerve axons *in vivo*. Individual data points represent sciatic nerve axons. **(C)** There is no difference in the mitochondrial motility index between young and old neurons (*i.e.*, total moving mitochondria/(total moving + stationary mitochondria)). **(D)** The relative proportions of anterograde and retrograde mitochondrial transport are not changed by age in the sciatic nerve, indicating that the reduction of both types of transport contributes to the overall transport decline (Figure 2D). Individual data points represent fields of view (FOV) imaged (1-3 axons/FOV). Data are shown as mean ± SEM. Mann-Whitney test (A-B), unpaired student’s t-test (C), two-way ANOVA with Tukey’s multiple comparisons test (D). ** p < 0.01; **** p < 0.0001.

**Supplementary Figure 3**


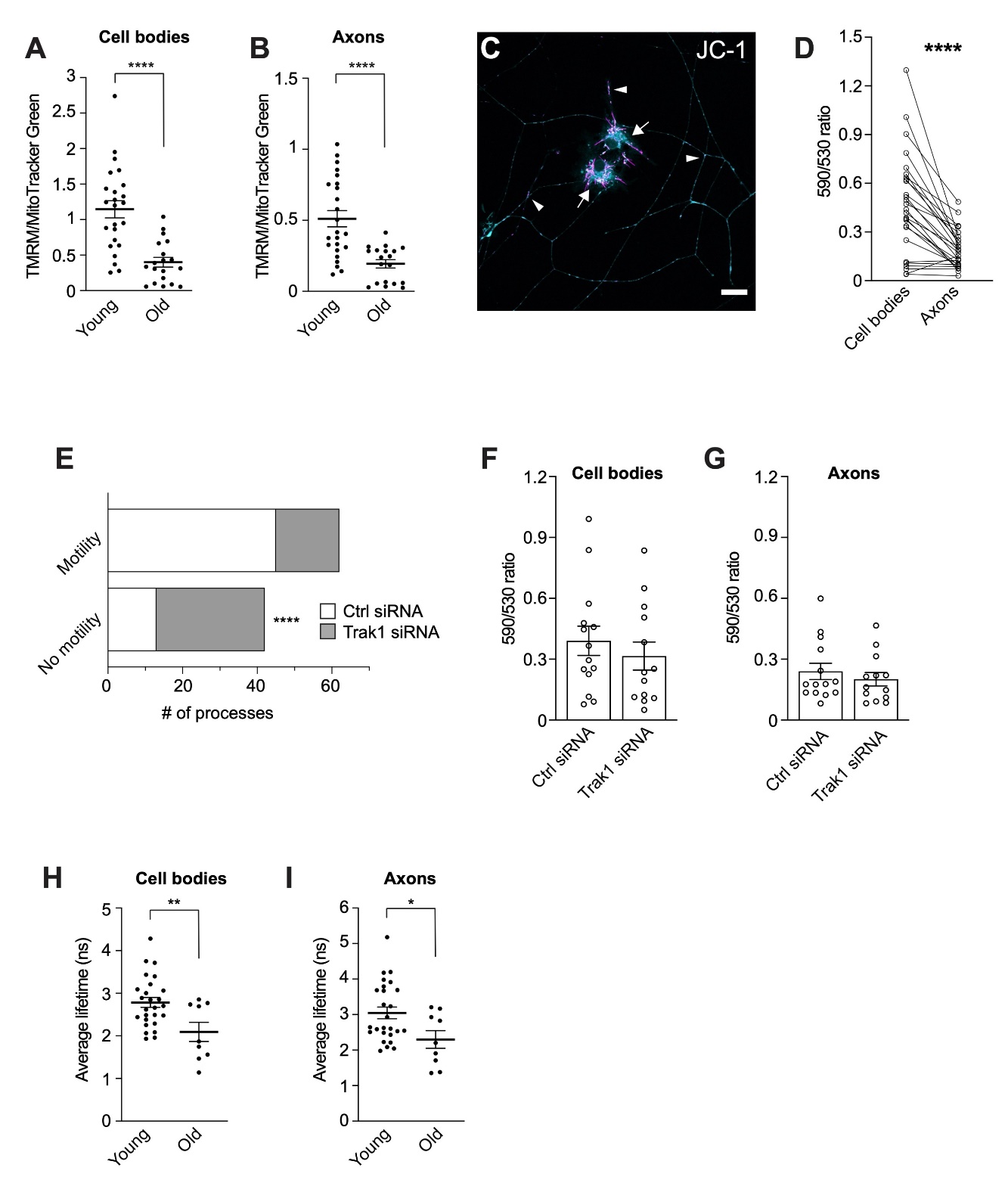


**Figure S3**

**(A-B)** Quantification of the ratio between TMRM and MTG in neuronal cell bodies (A) and axons (B) indicates that the ΔΨ_m_ declines during ageing in both compartments. **(C)** DRG neurons stained with the JC-1 vital dye and pseudocoloured in cyan and magenta (~ 530 and ~ 590 nm emission, respectively). Arrows, somal compartments; arrowheads, axons. Note the higher polarisation of somal mitochondria compared to axonal mitochondria, indicated by widespread magenta, corroborating the data in Fig. 3A-G. Scale bar: 20 μm. **(D)** Quantification of the 590/530 JC-1 ratio from young DRG neurons indicates that the axonal mitochondria display reduced ΔΨ_m_ compared with cell body mitochondria. Data points represent the cell bodies and axons imaged, with each neuron providing both cell body and axons for paired analysis. **(E)** Increased number of DRG neuron axons displaying no motile mitochondria after Trak1 RNAi. A process is scored as displaying motility if at least two mitochondria are motile within 1 min of imaging. Number of axons analysed are 58 (Ctrl siRNA) and 46 (Trak1 siRNA). **(F-G)** Trak1 RNAi does not significantly change the 590/530 JC-1 ratio in the cell bodies (F) or axons (G) of DRG neurons, suggesting the ΔΨ_m_ is not affected by this manipulation. Data points represent individual cell bodies and axons, respectively. Each neuron analysed provides both cell body and axons to the analysis. DRGs for JC-1 quantifications were obtained from 3 young (P102-105) male mice. **(H-I)** Mitochondrial viscosity decreases during age both in the cell bodies (H) and axons (I) of DRG neurons. Data points represent cell bodies and axons imaged. Data are shown as mean ± SEM. Unpaired student’s t-test (A, F, H), Mann-Whitney test (B, G, I), paired student’s t-test (D), Fisher's exact test (E). * p < 0.05; ** p < 0.01; **** p < 0.0001.

**Supplementary Figure 4**


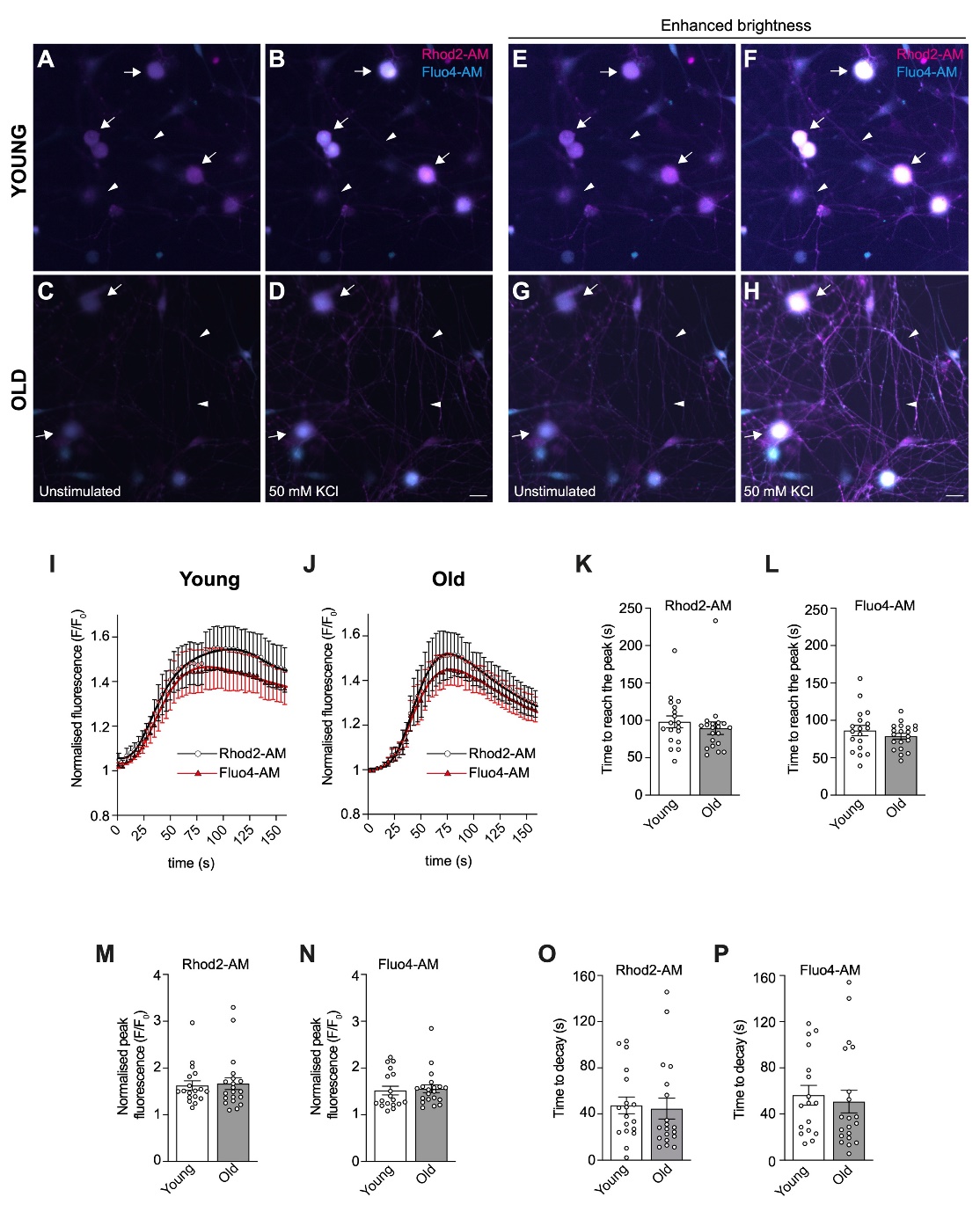


**Figure S4. Mitochondrial and cytosolic calcium uptake are not significantly affected by age in cultured DRG neurons**

**(A-H)** Representative images of DRGs cultured before (A,C,E,G) and after (B,D,F,H) stimulation with 50 mM KCl. Cells are co-stained with Rhod2-AM (magenta) and Fluo4-AM (cyan) for measuring the fluorescent signal of mitochondrial and cytosolic Ca^2+^, respectively. Neuronal depolarisation by KCl increases calcium uptake in cell bodies (arrows) and axons (arrowheads). Panels A-D are shown with enhanced brightness in E-H to highlight the response after treatment. Scale bars: 20 μm. **(I-J)** Traces indicate the average Rhod2-AM (black, circles) and Fluo4-AM (red, triangles) fluorescence intensity values in 18 young (I) and 20 old (J) neurons at individual time points (circles and triangles, respectively) normalised to the average fluorescence value before KCl stimulation (time 0). **(K-L)** Time to reach the peak, **(M-N)** normalised response peak and **(O,P)** decay time for cytoplasmic (Fluo4-AM) and mitochondrial (Rhod2-AM) calcium. Mann-Whitney tests (K,M-P) and an unpaired student’s t-test with Welch’s correction (L) showed no difference between conditions. DRGs were obtained from 3 young (P58-93, 3 males) and 4 old (P560-676, 1 female, 3 males) mice.

**Supplementary Figure 5**

**
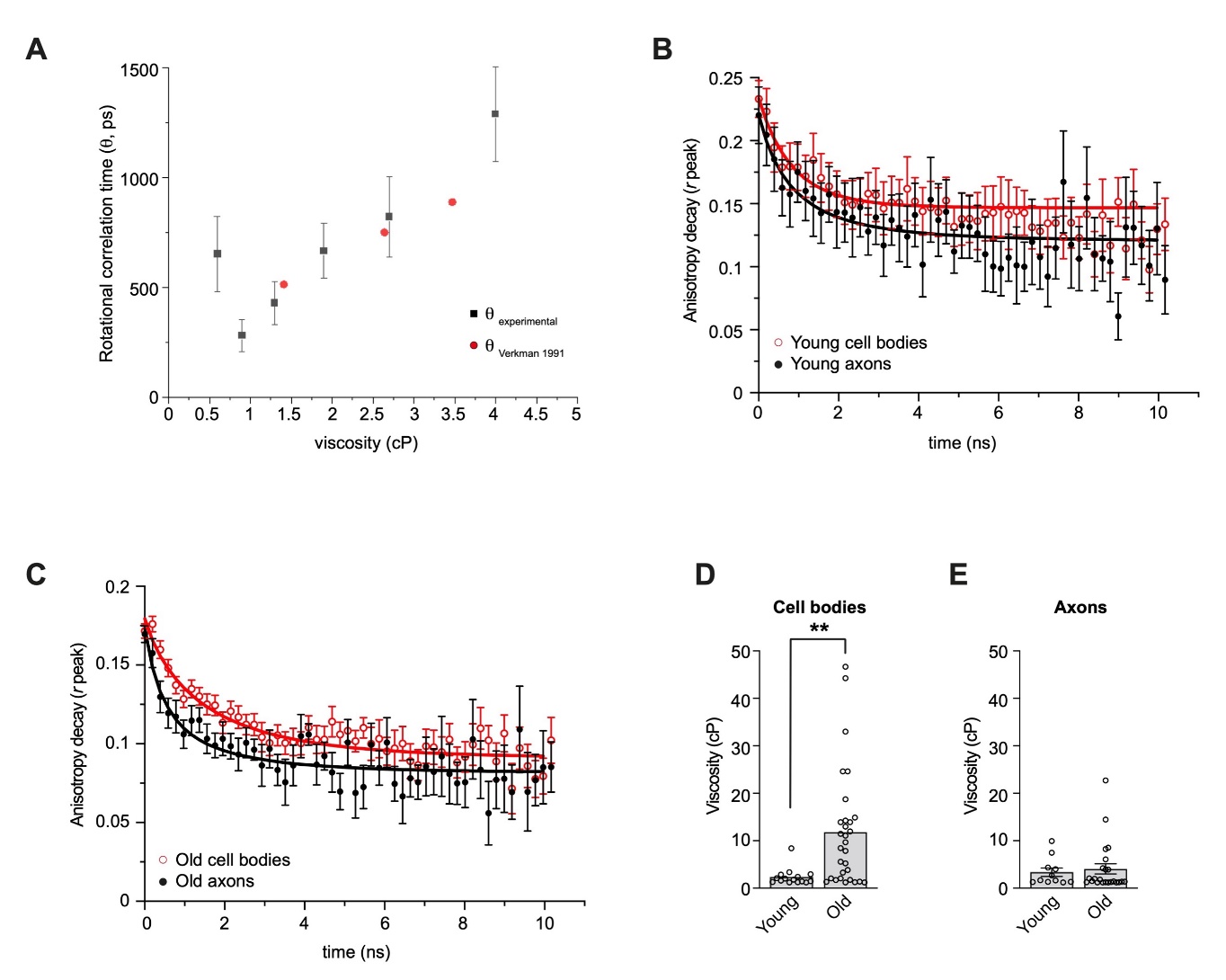
**

**Figure S5**

**(A)** Calibration curve of BCECF rotational correlation time (θ) calculated in accordance with the Stokes-Einstein-Debye equation. The experimental data were compared to historical data for BCECF θ as a function of viscosity ([Verkman et al., 1991](#_ENREF_80)) and showed good agreement. **(B-C)** Fluorescence anisotropy decay and fit (see Methods, equation 3) of BCECF fluorescence obtained from perpendicular and parallel decays (according to equation 1, see Methods) from cell bodies and axons of young (B) and old (C) DRG neurons. Red and black lines depict exponential decay fit for cell bodies and axons, respectively. The anisotropy decay of young cells (B) is not significantly different between compartments, leading to similar viscosity values (Fig. 4D). The slower decay of cell bodies compared to axons in old DRGs is indicative of higher viscosity, as shown in Fig. 4E. Circles, individual time points. **(D-E)** Cytoplasmic viscosity increases during age in the cell bodies (D) but not axons (E) of DRG neurons. Circles depict cell bodies and axons imaged. Data are shown as mean ± SEM. Mann-Whitney test (D-E). ** p < 0.01.

**Supplementary Figure 6**


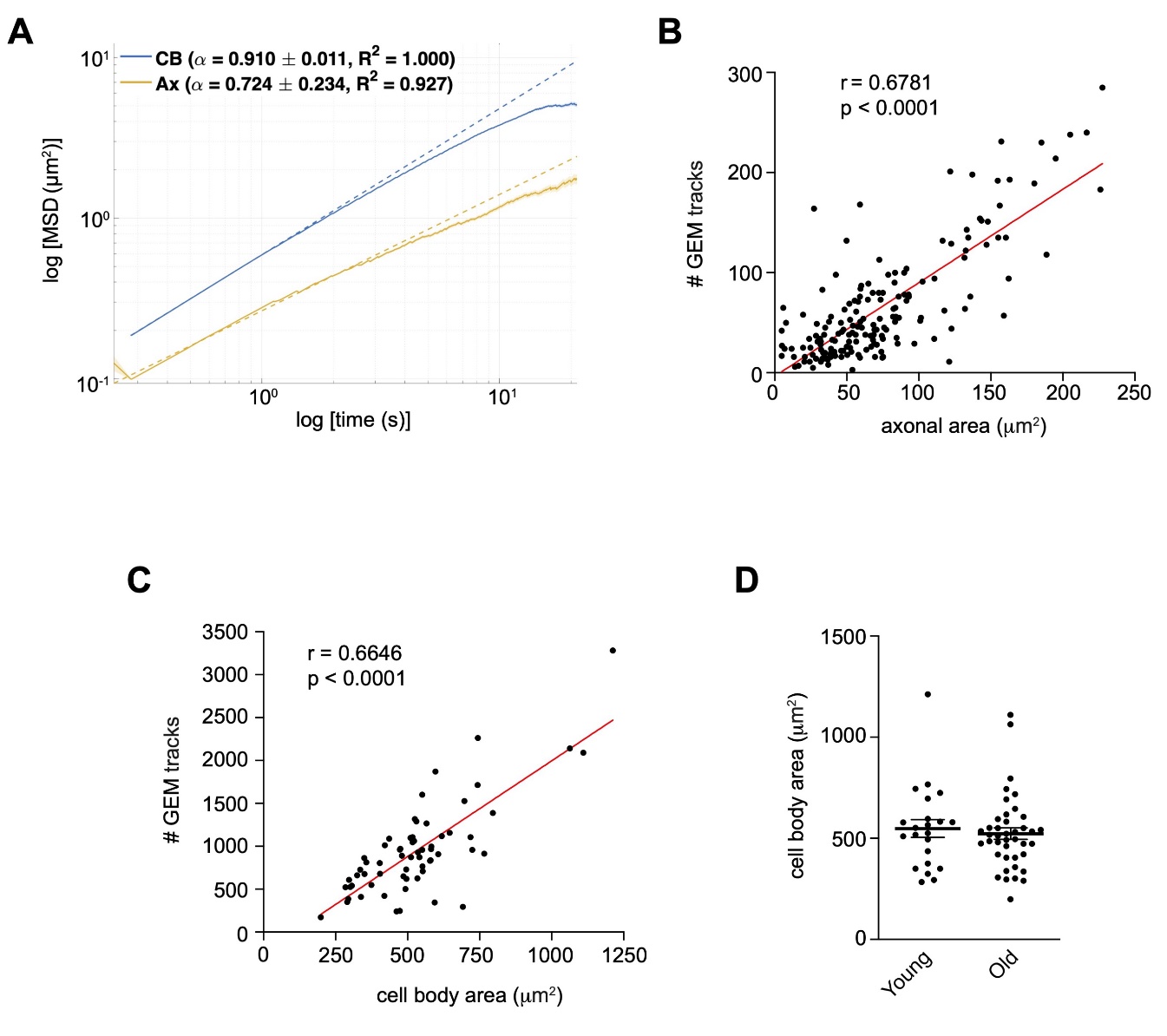


**Figure S6**

**(A)** Combined MSD of GEM tracks­ in cell bodies (CB, blues) and axons (Ax, orange). Data are plotted as mean ± SEM in a log-log format. Dashed lines are curve fit with anomalous exponent of 0.910 ± 0.011 (CB) and 0.724 ± 0.234 (Ax), respectively. **(B-C)** Positive correlation between total number of GEMs tracks analysed and axonal (B) and cell body (C) areas, suggesting that GEMs equally cover the full extent of the neuronal cytoplasm, independently of cell size. Circles represent individual axons (B) and cell bodies (C). Red lines, linear regression fit. Spearman rho test. **(D)** There is no significant difference between the mean area of the young and old cell bodies, indicating that cell size is unlikely to affect viscosity and diffusiveness. Circles represent individual cell bodies. Data are shown as mean ± SEM. Mann-Whitney test (p = 0.5148).

**Supplementary Figure 7**


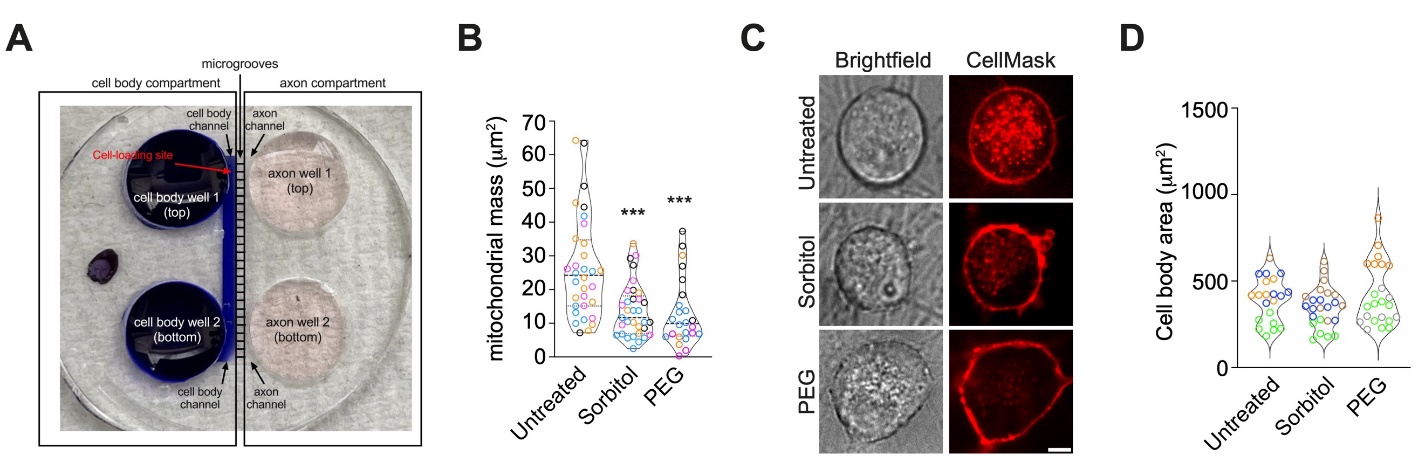


**Figure S7**

**A)** Microfluidic chamber (MFC) showing reliable fluidic separation between the cell body and axonal compartments. A blue dye was applied to the somal compartment at a lower volume than the connecting axonal compartment and incubated at 37°C for up to 5 h. Visual inspection confirmed that there is no dye leakage against the volume gradient. The same outcome was achieved after staining the axonal compartment. **(B)** Quantification of mitochondrial mass (see Methods) shows strong decline in sorbitol and PEG-treated DRGs. Datapoints in the violin plots represent individual microgrooves with each colour indicating a different animal (4 females, P45-86), Kruskal-Wallis with Dunn’s multiple comparisons test. **(C)** Representative images of DRG cell bodies that have been left untreated (top panels) or treated with sorbitol (middle panels) or PEG (bottom panels). The outline of the cell body defined by CellMask staining was used as a reference for the quantification of cell body area in (D). Scale bar: 5 μm. **(D)** Quantification of the DRG cell body area (see Methods) shows no significant change across conditions, unpaired t-tests with Welch’s correction (untreated vs sorbitol) and Mann-Whitney test (untreated vs PEG). Datapoints in the violin plots represent cell bodies from different MFCs, with each colour indicating a different animal (3-4 females, P45-86). *** p < 0.001.

**Supplementary** **Table 1**

| **A. Young** | | | |
| --- | --- | --- | --- |
| Velocity (μm/s) | | | |
| Anterograde | | Retrograde | |
| *In vivo* | *In vitro* | *In vivo* | *In vitro* |
| 0.63 (2.55),  n = 1018 | 0.45 (2.94),  n = 236 **** | 0.58 (2.01),  n = 428 | 0.44 (2.98),  n = 141 **** |
| Displacement (μm) | | | |
| Anterograde | | Retrograde | |
| *In vivo* | *In vitro* | *In vivo* | *In vitro* |
| 11.86 (64.92),  n = 1018 | 11.73 (83.80),  n = 236 | 11.95 (64.26),  n = 428 | 9.02 (72.47),  n = 141 ** |

| **B. Old** | | | |
| --- | --- | --- | --- |
| Velocity (μm/s) | | | |
| Anterograde | | Retrograde | |
| *In vivo* | *In vitro* | *In vivo* | *In vitro* |
| 0.69 (1.7),  n = 507 | 0.51 (4.41),  n = 183 **** | 0.67 (2.17),  n = 233 | 0.43 (2.85),  n = 137 **** |
| Displacement (μm) | | | |
| Anterograde | | Retrograde | |
| *In vivo* | *In vitro* | *In vivo* | *In vitro* |
| 13.31 (65),  n = 507 | 8.62 (95.13),  n = 183 **** | 13.58 (65),  n = 233 | 7.21 (85.50),  n = 137 **** |

**Table S1**

Median mitochondrial velocities and displacements are significantly higher in the sciatic nerve axons (*in vivo*) compared to DRG axons (*in vitro*) in all conditions except for anterograde displacement in young samples (A) where no difference was detected. Values are medians with, in brackets, maximum speeds and maximum displacements. n = number of mitochondria, Mann-Whitney test. ** p < 0.01; **** p < 0.0001. The data suggest that, while mitochondria are able to reach faster maximum velocities *in vitro*, on average they move more slowly *in vitro* than *in vivo*. Note that the displacement is calculated over 65 μm and 100 μm of axonal tract in the sciatic nerve and cultured DRGs, respectively, suggesting the calculated values may underestimate the significance between *in vivo* and *in vitro* displacements.
